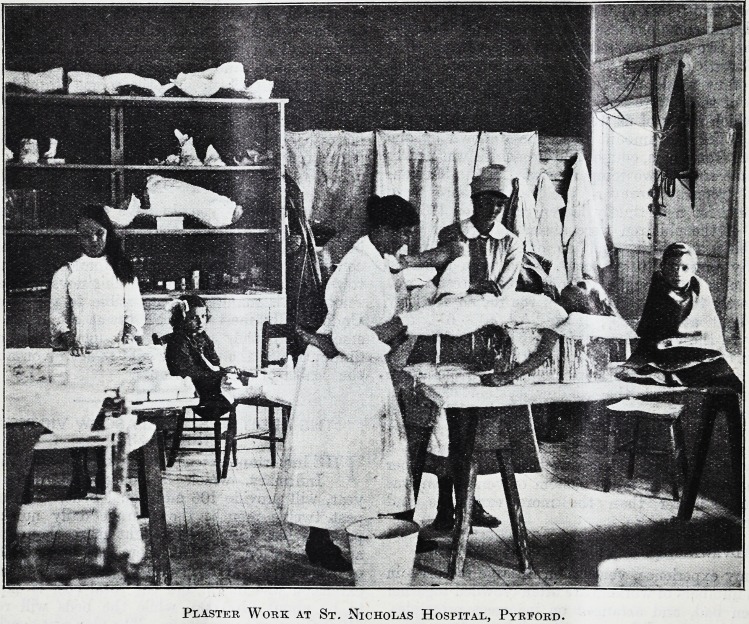# The Care and Cure of Cripple Children

**Published:** 1924-03

**Authors:** 


### THE CARE AND CURE OF CRIPPLE CHILDREN.

### DEVELOPMENT OF THE HOSPITAL SCHOOL.

UNDER the title of " The Care and Cure of Cripple Children," the Central Committee for the Care of Cripples has issued through Messrs. John Wright and Son, of Bristol (2s. 6d.), a description of their scheme for the welfare of cripple children by C. R. Girdlestone, F.R.C.S., together with a year book of Hospital schools and other institutions for these children, compiled by Mrs. Hey Groves.

In his Preface Sir Robert Jones writes :—

The objects of the committee, acting in association with the Council of Infant and Child Welfare, are :—To promote a national scheme for complete provision of treatment and education for physically defective children throughout the country. To help any local authority or voluntary organisation desirous of making better provision for these children. To provide a central bureau of information on these matters. To act as a central co-ordinating body. Outside of these various agencies it is a matter of profound satisfaction that the Board of Education and the London School Board are awakening to the racial and civic problem of the cripple. What can now be done, however inadequate, to make paralysis and deformity no longer insuperable bars to useful citizenship is not a dream but a reality, which is being evermore fully demonstrated. In 1904 the first residential cripple school was founded at the Heritage, Chailey, Sussex. There are now about sixty day schools and thirty-five residential schools for the education of the physically defective. But education alone was not enough! The Education Act of 1918 extended further help and imposed upon local authorities the duty of looking after the health as well as the education of physically defective children. This helped the development of the hospital school.

There are now some 80,000 to 100,000 crippled children in this country, and with these children, as Dr. Girdlestone points out, the general hospitals have not been able to deal effectively. Each district should initiate its own unit—" surgeons, nurses, voluntary workers, hospital and clinic committees, push off together, a picked company" :—

Voluntary effort must now as always lead the way, make the adventure, supply the expert knowledge, and give a convincing demonstration. In several counties first-class open-air hospital schools already exist, but lack intimate association with the district surrounding them by means of clinics. At present some of these hospitals take patients from all over England, with inevitable delay in admission and lack of orthopædic supervision after discharge. In such places the association of each hospital with a suitable district surrounding it is alone needed. A great beginning has been made. In Shropshire, Cheshire, Staffordshire, Lancashire, Westmorland, Cumberland, Herefordshire, Oxfordshire, Berkshire, Buckinghamshire, Hertfordshire, Wiltshire and Surrey this joint work of hospital and clinic exists or is coming into being. It is not a matter of who will be first, but of who will be last, and—" the devil take the hindmost."

PLASTER WORK AT ST. NICHOLAS HOSPITAL, PYRFORD.

A copy of the book is being sent to every member of Parliament.

## Figures and Tables

**Figure f1:**